# Impact of Chronic Kidney Disease on Brain Structure and Function

**DOI:** 10.3389/fneur.2022.797503

**Published:** 2022-02-25

**Authors:** Emily J. Steinbach, Lyndsay A. Harshman

**Affiliations:** ^1^Department of Radiation Oncology, Carver College of Medicine, University of Iowa, Iowa City, IA, United States; ^2^Division of Nephrology, Dialysis, and Transplantation, University of Iowa Stead Family Children's Hospital, Iowa City, IA, United States

**Keywords:** chronic kidney disease, magnetic resonance imaging (MRI), pediatric chronic kidney disease, neurocognition and behavior, brain structural abnormalities, neuroimaging, disease and development

## Abstract

Chronic kidney disease (CKD) affects more than 37 million American adults. Adult-onset CKD is typically attributed to acquired comorbidities such as aging, type II diabetes, and cardiovascular disease. Conversely, congenital abnormalities of the kidney and urinary tract are the most common cause of CKD in children. Both adult and pediatric patients with CKD are at risk for neurocognitive dysfunction, particularly in the domain of executive function. The exact mechanism for neurocognitive dysfunction in CKD is not known; however, it is conceivable that the multisystemic effects of CKD—including hypertension, acidosis, anemia, proteinuria, and uremic milieu—exert a detrimental effect on the brain. Quantitative neuroimaging modalities, such as magnetic resonance imaging (MRI), provide a non-invasive way to understand the neurobiological underpinnings of cognitive dysfunction in CKD. Adult patients with CKD show differences in brain structure; however, much less is known about the impact of CKD on neurodevelopment in pediatric patients. Herein, this review will summarize current evidence of the impact of CKD on brain structure and function and will identify the critical areas for future research that are needed to better understand the modifiable risk factors for abnormal brain structure and function across both pediatric and adult CKD populations.

## Introduction

Neurocognitive deficits have been well-described in both the adult and pediatric chronic kidney disease (CKD) and end-stage kidney disease (ESKD) populations (1, 2). These deficits are associated with longer duration of kidney disease (1, 3, 4), metabolic acidosis (5, 6), proteinuria/microalbuminuria (1, 7, 8), anemia (1, 9, 10), and hypertension (11, 12). Even subtle neurocognitive deficits have broad impacts on quality of life, as they contribute to poorer high school graduation rates and long-term underemployment in the adult CKD population (13). The cognitive complications of CKD may be linked to an aberrant “kidney-brain axis” whereby decreases in kidney function, CKD-associated sequelae (including cardiovascular disease), and concomitant inflammatory milieu all negatively impact the brain, leading to increased risk of cognitive impairment in parallel with CKD progression (14–16).

Unfortunately, our understanding of the neurobiology of cognition in CKD is limited because of a lack of concurrent neuroimaging and neurocognitive assessment. Neurodevelopment is a dynamic process occurring throughout the human lifespan, with the most rapid neurodevelopmental changes occurring in childhood and adolescence (17)—specifically, reductions in cortical gray matter (e.g., dendritic pruning) and concomitant white matter (myelin) deposition (18). Our understanding of normal developmental processes in the context of chronic disease is limited. Use of magnetic resonance imaging (MRI) provides an opportunity to examine the brain structure, as it relates to CKD, in a noninvasive manner.

To reduce the burden of neurocognitive deficits within the CKD population, there is an urgent need for new approaches to patient care applied across CKD lifespan. Such approaches require understanding the effects of CKD progression and severity on both the adult and pediatric brain. In this review we will 1) address existing literature specific to the impact of CKD on brain structure and function and 2) discuss potential CKD-associated risk factors for abnormal brain structure and function in both pediatric and adult populations.

## How Does CKD Impact Brain Structure and Function?

### Neuroimaging in Adult CKD

More than 37 million adults in the United States are living with CKD and millions more are living with either undiagnosed CKD or with an increased lifetime risk of developing CKD. Diabetes and hypertension are the leading causes of CKD in adults, contributing to almost 66% of CKD cases in the United States (19). Cardiovascular disease remains the major cause of death for individuals with CKD.

Structural brain findings of adult patients with CKD–not receiving renal replacement therapy–have demonstrated the presence of cerebral atrophy, as well as decreased cerebral density in both white and gray matter. Poor kidney function has been associated with glomerular small vessel disease, and in the 2008 study by Ikram et al. (20) hemodynamic similarities were investigated between the kidneys and vascular beds of the brain by MRI. Here, impaired kidney function was associated with smaller brain volume, smaller deep white matter volume, and more white matter lesions (20). Other randomized trials, including the Systolic blood PRessure INterventional Trial (SPRINT), examined the effects of hypertension treatment on the structure of the brain in patients with and without CKD, with cognitive endpoints noted (21). The SPRINT study found that CKD patients who were on standard blood pressure treatments had increased risks of mortality and major cardiovascular events, demonstrated mild cognitive impairment, and showed small vessel ischemic disease (white matter lesions) by MRI.

White matter hyperintensities are noted more frequently within the adult CKD/ESKD population. These hyperintensities are often associated with increased cerebrovascular risk and may predict risk of stroke and dementia (22). Diffusion tensor imaging (DTI) is an MRI technique used to provide detailed images of the brain. MRI-DTI measures the rate at which water moves through the brain's white matter (23). While *macrostructural* MRI markers, such as white matter hyperintensities, correlate with reduced kidney function, loss of white matter *microstructural* integrity may be a more sensitive measure of white matter disease (24). White matter degeneration and white matter abnormalities may be attributed to several causes. First, the kidneys and brain share several perfusion pathways in which cardiovascular and hemodynamic deviances may damage both organs simultaneously. Other factors, including the hypertension seen in many CKD patients, may also play a role in the association between kidney function and white matter integrity (25). Finally, impaired kidney function can lead to increased circulating inflammatory factors. Proinflammatory factors decrease serum nitric oxide in the brain vasculature, and this could contribute to cerebral hypoperfusion, which, in turn, could lead to white matter damage as well (25).

Cerebral hypoperfusion has been implicated in neurodevelopmental disorders. Hemodynamic disturbances during CKD may play a role in the regulation of cerebral blood flow (26), potentially linking CDK to cognitive problems. An analysis based on the Rotterdam Study (27) found that lower estimated glomerular filtration rate (eGFR) is independently associated with lower cerebral blood flow (27). More recently, Lepping et al. (28) used arterial spin labeling to assess cerebral blood flow in a cohort of EKSD patients and age-matched controls. The authors' goal was identify kidney-associated brain changes following kidney transplantation. Here, after kidney transplant, cerebral blood decreased in ESKD patients to values comparable to controls. White matter integrity, as measured by fractional anisotropy and by mean diffusivity with MRI-DTI, also increased and decreased, respectively, post-kidney transplant (28). These measurements of white matter integrity taken after kidney transplant were comparable to controls. Taken together, these data suggest that proper kidney function is essential for regulation of blood flow to the brain and hemodynamic homeostasis.

### Neuroimaging in Pediatric CKD

In contrast to adult CKD, congenital anomalies of the kidney and urinary tract are the leading causes of CKD among children ages birth to 4 years, whereas systemic diseases, infection, and glomerular disease (e.g., focal segmental glomerular sclerosis) become the leading causes of kidney failure in the older pediatric population (29). Up to half of all children with congenital CKD will experience a decline in kidney function so severe as to require dialysis and eventually, a kidney transplant (30). Thus, children with congenital anomalies of the kidney and urinary tract are faced with a lifetime of CKD and of potential detrimental impacts on the developing brain.

Neuroimaging studies in the pediatric population have focused mainly on children with moderate to severe CKD (including dialysis and transplant populations). The effect of kidney disease in these children introduces many confounding factors that affect brain function, such as uremia. Another limitation of imaging studies in older children is that these studies cannot provide direct information about the key stages of brain development that occur from birth to 4 years of age. These issues signal a critical gap in our understanding of the kidney-brain axis during periods of peak neurodevelopment.

Cystic kidney diseases are some of the leading causes of early-onset inherited kidney disorders. These diseases are often characterized by enlarged kidneys with multiple cysts and progressive kidney impairment. Autosomal dominant and autosomal recessive polycystic kidney disease, as well as Meckel's syndrome, are a few examples of renal ciliopathies seen in the pediatric population. Cysts develop due to uncontrolled epithelial cell proliferation, growth, and altered cell polarity—events that occur downstream of abnormal cilia-dependent signaling. Due to the early onset of renal ciliopathy-induced CKD and concomitant development of severe hypertension, it is thought that ciliopathies are a risk factor for neurocognitive dysfunction secondary to CKD (31). The spectrum of neurocognitive deficits ranges from relatively benign (akin to that seen in polycystic kidney diseases) to more progressive deficits in neurocognition (such as that seen in Joubert syndrome). One cross-sectional, control-matched analysis, which involved the Chronic Kidney Disease in Children (CKiD) cohort, compared a group of autosomal recessive polycystic kidney disease patients with mild-to-moderate CKD patients with respect to intellectual functioning, academic achievement, attention regulation, executive functioning, and behavior (31). No differences were observed between these two disease cohorts; however, further investigation into the potential effects of renal ciliopathies on neurocognition in the pediatric population is needed.

The majority of published pediatric CKD studies evaluating volumetric brain structure have used computerized tomography (CT) imaging data obtained in a clinical setting, rather than quantitative MRI data obtained as a part of a specific research focus (19). Brain atrophy is well-described in the early pediatric nephrology literature (prior to 1990): up to 60% of patients had atrophy that was not clearly related to etiology of disease or CKD-associated sequelae such as hypertension (32). Qualitative CT imaging also provides evidence for global cerebral atrophy, silent white matter infarcts, and ventriculomegaly in advanced pediatric CKD (19). In other studies, lower cerebral density (33, 34) and ventriculomegaly secondary to brain atrophy (35) were found to be associated with the need for pediatric hemodialysis (especially duration), but not with peritoneal dialysis (36).

**Figure 1:**
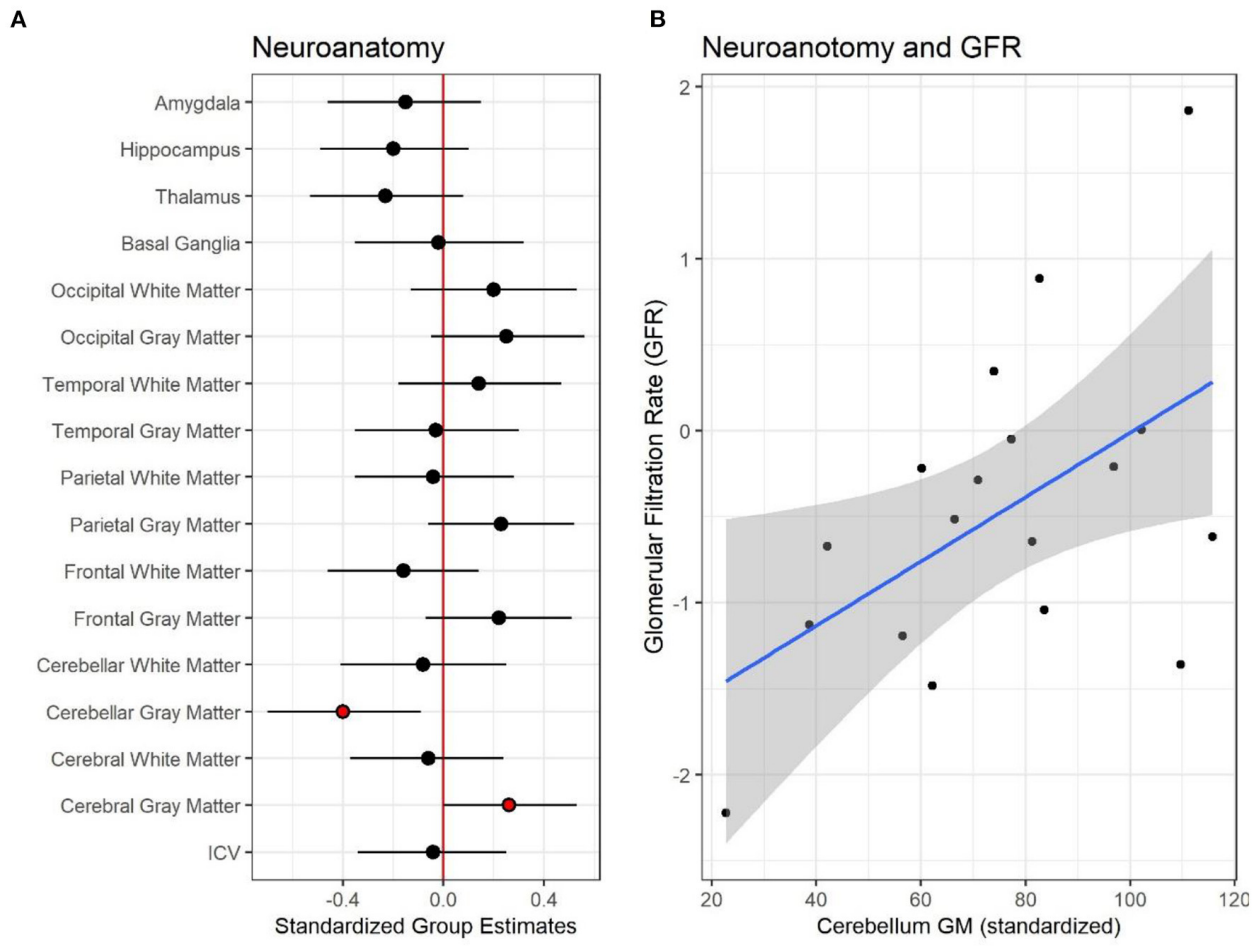
Neuroanatomical differences between controls and patients with pediatric chronic kidney disease CKD. **(A)** Shows the standardized group estimates (x-axis) and 95% confidence limits of the estimates for each of the regions of interest (ROI) included in the analysis (y-axis). Estimates are adjusted for age, socioeconomic status (SES) and maternal education. The red (vertical) line marks 0, or no significant effect of group on ROI. Red circles mark significant group estimates. **(B)** Shows the relationship between estimated glomerular filtration rate, eGFR, (x-axis) and standardized cerebellum gray matter volume (y-axis) in the CKD group. Reproduced with permission from Solomon et al. (39).

**Figure 2:**
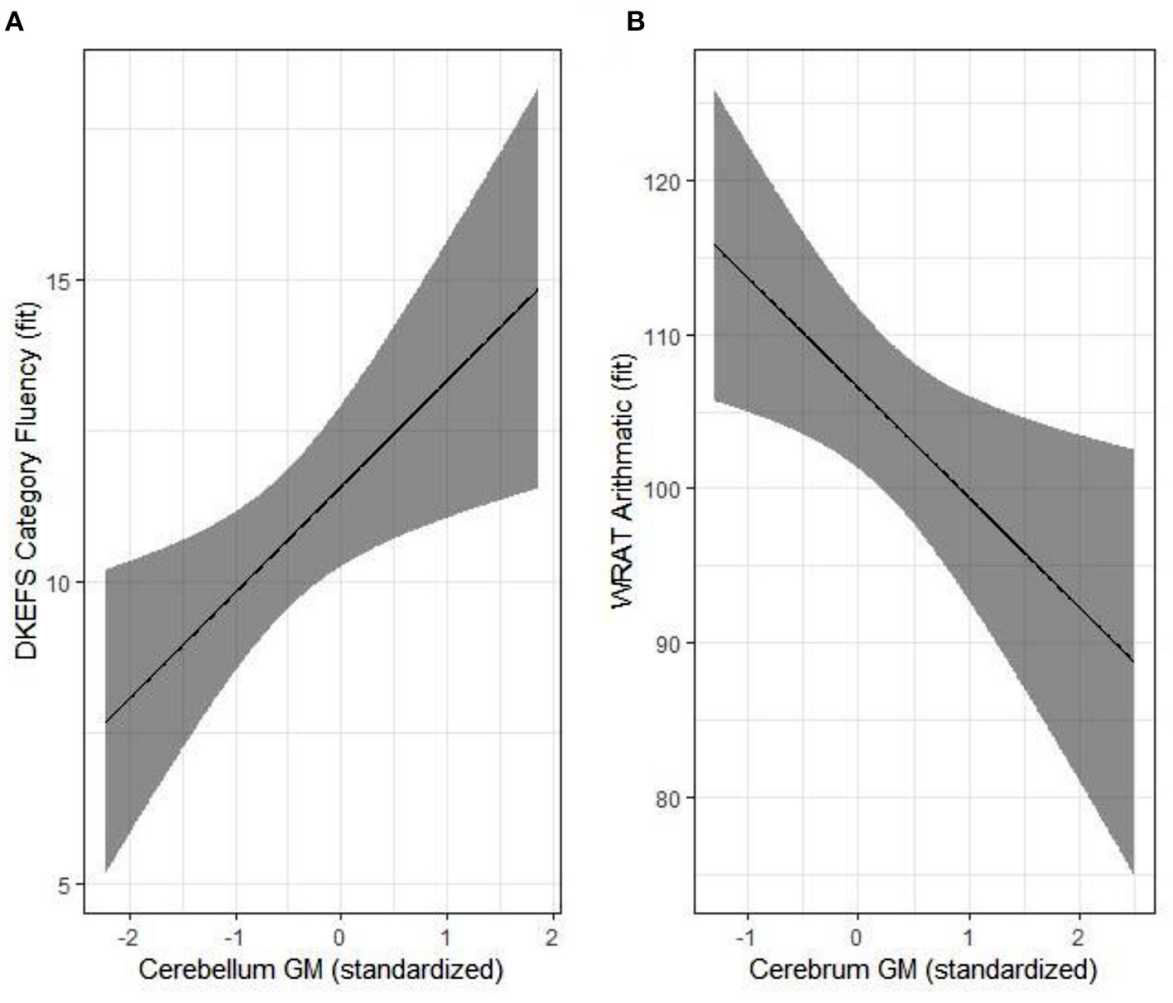
Structure-function relationships in the pediatric chronic kidney disease (CKD) group. Significant associations are illustrated for cerebellum gray matter and category fluency **(A)** and cerebrum gray matter and arithmetic **(B)**. Results were adjusted for age, parental SES and maternal education. Reproduced with permission from Solomon et al. (39).

To date, there are only four published studies that have utilized quantitative, research-based MRI sequences to evaluate the brain in CKD (26, 37–40). Hartung and colleagues (41) used MRI to examine brain structure in 85 patients aged 8–25 with CKD, encompassing a mix of pre- and post-transplant patients (37). The authors reported subtle, cortical gray matter abnormalities; however, their findings were significant only in unadjusted models and did not persist in models adjusted for age and gender. In this study, observed volumetric brain differences were more prominent among the kidney transplant recipients compared to those with pre-transplant CKD. More recently, our group has observed significant reductions in overall cerebellar gray matter volume and unexpectedly, an increase in cortical (cerebral) gray matter volume among children ages 6–16 years old with early-stage CKD, relative to controls (39). The degree of cerebellar volume reduction was associated with estimated glomerular filtration rate (Figure 1). Volumetric reduction in the cerebellar gray matter was also associated with poorer performance in tests of executive function. The volumetric increase in the cerebral gray matter was associated with poorer mathematics performance (Figure 2).

CKD is a known risk factor for cerebrovascular disease, with white matter being particularly susceptible to the effects of altered vascular tone. Matsuda et al. (38) evaluated the impact of CKD on brain white matter within a cohort of 49 children, including 29 children with CKD of varying stages, ranging from pre- to post-transplantation. Diffusion tensor imaging was used to compare white matter microstructure in CKD patients compared to controls.

Patients with CKD were found to have abnormal white matter microstructure (specifically, within the anterior limb of the internal capsule), as reflected by decreased white matter fractional anisotropy and increased mean diffusivity and radial diffusivity. Within the sample, 21% of CKD participants had evidence of focal and multifocal white matter injuries compared to healthy controls. No neurocognitive data were obtained in this study; thus, the clinical significance of these white matter findings remains unclear.

**Figure 3:**
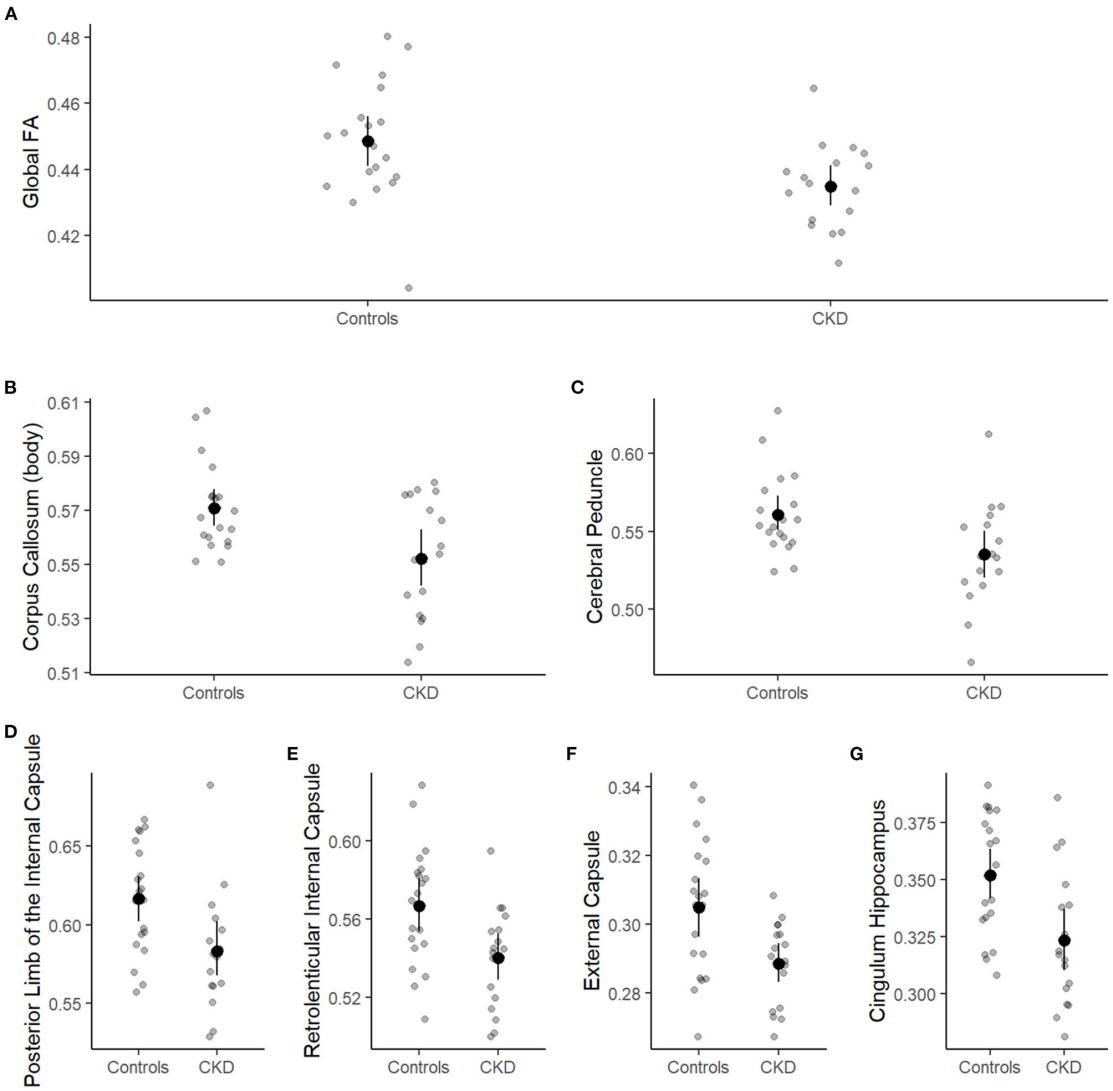
Group differences in global and regional white matter fractional anisotropy. Patient group (case or control) is shown on the x-axis and white matter fractional anisotropy on the y-axis. Individual observations are represented by small, gray circles. Means and 95% confidence limits of the means are shown for each group. **(A)** Shows the global fractional anisotropy, and **(B–G)** show white matter fractional anisotropy for the corpus callosum, cerebral peduncle, posterior limb of the internal capsule, retrolenticular internal capsule, external capsule, and cingulum hippocampus, respectively.

Recently, our group identified global abnormalities in white matter microstructural integrity in CKD patients compared to controls. The global decrease in white matter fractional anisotropy was driven by regional reductions within the body of the corpus callosum, cerebral, cingulum (hippocampus), and posterior limb of the internal capsule (Figure 3). Despite these significant differences in white matter integrity, we found no significant association between the neurocognitive abilities of CKD patients and white matter fractional anisotropy. Likewise, there were no CKD-associated medical variables that emerged as predictors for decreased white matter integrity.

Cerebral blood flow, which is a reliable measure of cerebrovascular integrity, can be quantified using arterial spin labeling MRI. Based on data from the Neurocognitive Assessment and Magnetic Resonance Imaging Analysis of Children and Young Adults with Chronic Kidney Disease (NiCK) study cohort (41), Liu et al. (26) found, unexpectedly, that global cerebral blood flow is higher in children with CKD compared to healthy controls (41). These findings are in line with adult data showing increased global cerebral blood flow, again as determined using arterial spin labeling sequences (42). Since increases in cerebral blood flow are associated with reduced hematocrit level in children with CKD (26), it has been hypothesized that the higher cerebral blood flow reflects a physiological compensation for the chronic anemia typically associated with advanced CKD.

## Disease or Development: Delineating Risk Factors for Brain Abnormalities in the Setting of CKD

Cognitive deficit in CKD has been linked to a variety of mechanisms associated with decreased kidney function, including concomitant uremia, proteinuria, anemia, metabolic acidosis, and cardiovascular disease (43). These CKD-associated medical sequelae become more prominent in CKD stage 3 and beyond (eGFR <60 ml/min/1.73 m^2^). Elevated blood urea nitrogen (uremia) is a CKD-associated comorbidity that often appears together with cognitive problems. Neurons, such as the noradrenergic and serotonergic neurons responsible for sleep/wake cycles and motor control, as well as the acetylcholinergic neurons responsible for memory, may be particularly sensitive to high levels of uremic milieu (44). Alterations in the monoaminergic neurons may contribute to the transient development of cognitive impairment that is seen in patients with advanced CKD requiring dialysis (44). Although uremia is commonly invoked as a primary etiology for cognitive deficit in CKD, a clear neuroimaging correlate to link changes in the brain with symptomatic uremia is lacking. One recent case series evaluated MRI scans from patients with clinically documented uremic encephalopathy and showed bilateral basal ganglia lesions in the majority of images (45). However, since no neurocognitive phenotype was described in this study, no inference can be drawn as to whether the lesions noted were associated with cognitive deficit.

Metabolic acidosis may play a direct role in cognitive impairment in adults with CKD. Animal data suggest that metabolic acidosis perpetuates neuronal dysfunction by upregulation of excitatory synapses on gaba-aminobutyric acid-ergic neurons (44, 46). The SPRINT-MIND [Systolic Blood Pressure Intervention Trial Memory and Cognition IN Decreased Hypertension (21)] cohort assesses adults with hypertension and includes adults with CKD. Data from this study showed that decreases in serum bicarbonate were independently associated with lower performance on tests of executive function (5). Harshman et al. (6) evaluated the impact of metabolic acidosis in relationship to blood pressure variability in pediatric CKD patients using the CKiD cohort. The researchers found that the effect of increased blood pressure variability on executive function was attenuated in the setting of higher serum bicarbonate levels.

Hypertension (HTN), a CKD-associated comorbidity, can affect the severity and course of cerebrovascular disease. Consequently, HTN is a potentially modifiable risk factor for cognitive defects in both pediatric and adult CKD populations. As noted previously, the SPRINT study is the largest intervention study to date looking at the effect of intensive blood pressure control on cardiovascular outcomes among persons at high risk for cardiovascular disease. The SPRINT trial demonstrated a role for intensive systolic blood pressure control (goal of <120 mm Hg) in the reduction of probable dementia within an adult CKD subgroup (21). Importantly, data from the SPRINT-MIND sub-analysis found no detrimental effect of intensive lowering of systolic blood pressure on brain perfusion or volumetric structure (47).

The neurocognitive deficits in the adult CKD population exacerbate comorbidities and contribute to a lower quality of life. The most frequently impaired cognitive domains in the adult CKD population include executive function, orientation, and attention (48). Multiple studies have reported increases in cognitive impairment with increasing age; however, even the younger kidney transplant candidate in the adult population has a relatively high burden of cognitive impairments compared to the average adult. The conclusions from Chu et al. (48) suggest that transplant centers consider screening kidney disease patients for global cognitive impairment throughout their clinical care regardless of age (48).

The association between hypertension, CKD, and cognition has received a significant amount of focus within the prospective CKiD cohort study. Lande et al. (12) evaluated data from children with mild to moderate CKD who have elevated blood pressure [i.e., systolic or diastolic blood pressure > 90th percentile for age (49)]. Children with elevated blood pressure were more likely to have lower nonverbal IQ than normotensive children. Within the analysis, it was also noted that the blood pressure index (i.e., the subject's blood pressure divided by the 95th percentile blood pressure for that subject's gender, age, and height) correlated inversely with nonverbal IQ, and this relationship was maintained even after controlling for demographic and disease related variables.

The neurocognitive deficits in the pediatric CKD population have also been assessed through batteries such as the Penn Computerized Neurocognitive Battery (50). This test revealed that children and young adults with CKD have lower accuracy in tests of complex cognition compared to their age-matched peers, as well as deficits in verbal reasoning, nonverbal reasoning, and spatial processing. Patients with CKD also had lower accuracy for attention but were found to have faster response times, possibly indicating greater impulsivity.

While unlikely to be the underlying cause of adult cognitive impairment, genetic factors may influence the pathogenesis of cognitive dysfunction in pediatric CKD patients. Both single-gene variants and copy number variants have been implicated as potential factors influencing cognitive deficit in pediatric CKD. Genomic differences associated with pediatric CKD were analyzed as part of the CKiD study (51); the aim was to determine whether genetic factors (in addition to, or perhaps rather than, renal impairment) were responsible for the subtle neurocognitive differences seen in pediatric CKD. Children with CKD-associated genomic disorders were found to score significantly poorer on all measures of intelligence and executive function compared to noncarriers (52).

Variation in the klotho gene has been associated with both an accelerated aging phenotype as well as development and progression of CKD (53, 54). Klotho is expressed in the brain, with high levels of mRNA expression in the choroid plexus, hippocampus, and cerebellar Purkinje cells (55, 56). The klotho gene is also highly expressed in the kidney, where Klotho acts as a coreceptor for fibroblast growth factor 23 (FGF23) in regulating calcium and phosphorus homeostasis (57). Homozygous klotho knockout mice and CKD subjects have similar phenotypes, suggesting that klotho dysfunction may contribute to CKD progression (54, 58). Additionally, Klotho-deficient mice demonstrate an accelerated aging phenotype characterized by neurodegeneration and cognitive deficits (59, 60). In both CKD patients and in mice lacking Klotho function, plasma FGF23 levels increase (61). Limited preclinical suggests that elevation of FGF23 is associated with abnormalities of hippocampal neural networks. While previous studies evaluating the effect of FGF23 on cognition have been equivocal in adults, data from the CKiD cohort suggests that a higher plasma FGF23 level is associated with higher cognitive impairment and lower performance in tests of executive function (55, 62).

Recent evidence suggests that there is crosstalk between the kidney and brain, and that this “kidney-brain axis” is sensitive to cellular oxidative stress and chronic inflammatory processes (14). The crosstalk, mediated by reactive oxygen species and inflammatory markers, may contribute to the high prevalence of cognitive impairment observed during the progression of CKD. Emerging data suggests that the metabolic interactions of the “kidney-brain axis” are likely mediated—at least in part–by the activities of hormetic processes and a phenomenon dependent on the severity of disease (63, 64). Oxidant-induced inflammatory pathways could be promising therapeutic targets for the protection of neurocognitive function in developing children who have CKD.

## Neuroimaging in CKD: Where Do We Go From Here?

In contrast to adult CKD populations, there is a paucity of systematic, quantitative neuroimaging studies in young children with CKD. The reasons for this are multiple. Certainly, there are clear challenges to obtaining quality images from non-sedated, young children. Furthermore, published pediatric CKD neuroimaging studies have relied on patient samples with heterogenous disease stage and etiology, making it difficult to pinpoint mechanisms of neurocognitive deficits across the lifespan. Additionally, there has been a stark lack of attention to the impact of CKD on early brain development: to date, only one neuroimaging study has been published that evaluated the brain in very young CKD patients (children younger than 8 years of age) (39).

The value of incorporating neuroscience-oriented analyses into pediatric CKD research is that this combined approach provides the clinical-translational tools needed to identify potential neurobiological mechanisms that bring about neurocognitive deficits in CKD children. This means moving beyond the use of clinical scans to describe brain structure in pediatric CKD; by their nature, these scans can highlight only a limited range of the potential neurobiological mechanisms. Multisite collaborations are necessary to address limitations related to small sample sizes and heterogeneity of neuroimaging research in pediatric CKD. And for these multisite neuroimaging initiatives to be effective, collaboration between nephrologists and neuroscientists is essential as well.

Neuroimaging research in the field of CKD requires tandem assessment of cognition and kidney disease sequelae, in order to identify patients who are at risk for neurocognitive deficits and also to learn how early brain changes relate to CKD progression. In comparison to the pediatric literature, the adult CKD-brain literature is robust and incorporates a comprehensive approach to the study of the brain. This should serve as an example for the field of pediatric nephrology to embrace when designing future CKD-brain studies. Neurocognitive difficulties emerge in early childhood, during early CKD, and signal a need for a greater understanding of how the developing brain is affected by this life-long, chronic disease process. Thus, longitudinal neurological assessment into adulthood is essential, and must continue through the period when CKD progresses to the point of requiring dialysis and/or kidney transplant. Future neuroimaging research is necessary to elucidate the neurobiological underpinnings of cognitive deficits in CKD.

## Author Contributions

The original manuscript, Impact of Chronic Kidney Disease on Brain Structure and Function, was ES and LH. Both authors had equal contribution to manuscript research, writing, and editing. All authors contributed to the article and approved the submitted version.

## Funding

This work was supported by R01DK128835 - active National Institutes of Health/NIDDK “Brain anatomical imaging and neurocognition in pediatric kidney disease (BRAIN KID)” (to LH).

## Conflict of Interest

The authors declare that the research was conducted in the absence of any commercial or financial relationships that could be construed as a potential conflict of interest.

## Publisher's Note

All claims expressed in this article are solely those of the authors and do not necessarily represent those of their affiliated organizations, or those of the publisher, the editors and the reviewers. Any product that may be evaluated in this article, or claim that may be made by its manufacturer, is not guaranteed or endorsed by the publisher.
